# Enhancing Lower Extremity Defect Coverage: High Viability Ultra-Thin Split-Thickness Skin Grafts Obtained from the Scalp

**DOI:** 10.3390/jcm12196109

**Published:** 2023-09-22

**Authors:** Alejandra Tomás-Velázquez, Javier Antoñanzas, Rafael Salido-Vallejo, Pedro Redondo

**Affiliations:** 1 Department of Dermatology, University Clinic of Navarra, 31008 Pamplona, Spain; atomasv@unav.es (A.T.-V.); rsalidov@unav.es (R.S.-V.); predondo@unav.es (P.R.); 2 School of Medicine, University of Navarra, 31008 Pamplona, Spain

**Keywords:** scalp, ultra-thin strip split-thickness skin graft, ulcer, tumor, DermaBlade, leg

## Abstract

Background: Repairing lower extremity defects presents challenges due to the scarcity of available local tissue. Skin grafting is a widely employed technique for addressing non-healing ulcers, improving the quality of life of patients and minimizing discomfort. However, using traditional donor sites, such as the thigh, can hinder mobility and result in noticeable scarring and pigmentation changes. Objectives: This study aims to assess the effectiveness of a novel approach utilizing autologous ultra-thin split-thickness skin grafts (STSGs) harvested from the scalp using a disposable, commercially available razor blade named DermaBlade. Methods: Fifteen patients (median age: 72 years, eight males and seven females) with diverse lower limb lesions, including carcinomas and ulcers of varying etiologies, were prospectively enrolled. Donor sites included the sideburn extending to the hairy temporal skin (nine cases) and hairy occipital skin (six cases). Ultra-thin skin strips (<0.2 mm thick) were obtained from the scalp through the use of the disposable flexible blade DermaBlade. The strips were positioned over the receptor area with no sutures in most cases and secured using dressings. A substantial majority of patients (90%) achieved successful graft take with no complications. Swift re-epithelialization occurred within a median of 12 days for the donor site and 24 days for the receptor site. No hair transfer or alopecic scars were noted. Conclusions: In contrast to traditional grafting methods, DermaBlade-assisted scalp grafting yields highly viable STSGs that adhere to wound beds without the need for sutures. Notable advantages of this technique encompass rapid wound healing, minimal complications, and superior cosmetic outcomes. Furthermore, it avoids scarring and alopecia, making it a promising approach for addressing lower extremity defects.

## 1. Introduction

Repairing complex wounds and surgical defects in the lower extremities is a challenging task due to the limited availability of suitable local tissue. Direct closure is often not possible and using local flaps can be problematic, particularly in elderly patients with compromised skin quality and restricted vascular supply [1,2]. In such cases, skin grafting has emerged as a common and effective technique for addressing non-healing ulcers, offering substantial improvements to patient quality of life. However, the specific indications for using skin grafts in lower extremity defects are still not well defined [3]. Even in cases where patients do not have underlying healing issues, lower extremity wounds typically require a prolonged healing period, making the wait for spontaneous healing impractical. This often leads to a significant number of medical visits accompanied by the corresponding healthcare expenses. The challenge becomes even more significant with ulcers, resulting from conditions such as venous insufficiency, diabetes mellitus, and rheumatoid arthritis, as they often do not respond well to extended and intensive conservative treatments [3]. Skin grafting can supply valuable benefits to these patients by providing support for non-healing tissue and alleviating associated pain. Various modalities exist and can be employed for cutaneous grafting [4,5].

Split-thickness skin grafts (STSGs), also known as partial-thickness skin grafts, involve using a piece of skin that includes the epidermis and the superficial portion of the dermis, mainly the papillary dermis. In contrast, full-thickness skin grafts encompass the epidermis, the entire dermis, and varying amounts of the subcutaneous tissue [6]. Thin grafts, such as STSGs, require less re-vascularization, thereby increasing the chances of graft survival and adherence. Consequently, they are ideal when the goal is to restore function with acceptable cosmesis, ensuring viability. Additionally, STSGs are further classified based on their thickness as thin (0.005 to 0.012 inches), medium (0.013 to 0.018 inches), or thick (0.019 to 0.028 inches) grafts [1,7]. The selection of an appropriate thickness for skin grafts in reconstructive surgery remains a challenge, with no clear gold standard established. The use of medium thickness STSGs should be the preferred choice whenever possible, as they typically provide a remarkable combination of graft survival and durability.

Another crucial decision in grafting is the careful selection of an appropriate donor site for harvesting. The choice of a suitable donor site is pivotal and involves measuring the dimensions of the wound, considering its length, width, and depth, to accurately determine the necessary amount of skin graft. Other factors to take into account encompass an efficient preparation, the optimal skin thickness and pigmentation, the complexity of harvesting, patient positioning, minimal bleeding, simple postoperative care, a low risk of infection, prompt wound healing, minimal disruption to rehabilitation, and aesthetic considerations, which can make the selection process a challenging task [1,5,7,8,9]. Typically, donor sites for STSGs can include the postauricular and subclavicular regions, the medial side of the upper arm, the back and buttocks, the inguinal ligament region, the anterolateral thigh and outer thigh, the popliteal fossa, the posterior calf or dorsal foot, and the plantar medial arch of the foot [1]. Nevertheless, selecting any of these locations as a donor site may lead to temporary mobility limitations and potential complications during healing, as well as noticeable scarring [8,9]. For example, described complications associated with harvesting from the lower limb (the thigh being the most frequent donor site employed) include severe pain, discoloration, hypertrophic scar formation, itching, deformity, reduced mobility, and the potential development of secondary deep vein thrombosis [10,11,12]. Interestingly, in our experience, the use of hairy scalp skin as the donor site offers several advantages, including rapid wound healing and low risk of complications, with excellent cosmetic results [10,11,12,13,14,15,16,17].

Finally, regarding the surgical instruments, a variety of tools have been used over time, including Catlin, Thiersch’s, Humby, or Watson grafting knives, as well as both manual and electric dermatomes [18,19]. However, in the current study, we selected and employed the disposable, commercially available DermaBlade (Personna DermaBlade, American Safety Razor Company, Cedar Knolls, NJ, USA) to obtain STSGs from the scalp. This blade is commonly used for performing shave biopsies and creating thin strips, offering potential advantages when compared to conventional scalpels or dermatomes due to its sharpness and flexibility.

Thus, to validate and illustrate this theory and to evaluate the efficacy and safety of our approach for repairing lower extremity defects, utilizing the scalp as a donor site and employing the DermaBlade as the cutting tool, we conducted a clinical prospective study, including patients with surgical defects and leg ulcers who received treatment at our center. The main objective was to assess the potential advantages offered by the innovative aspects of the proposed method.

## 2. Materials and Methods

### 2.1. Study Population and Data Collection

A longitudinal prospective follow-up study was conducted at the University Clinic of Navarra in Pamplona, Spain. Between March 2021 and May 2022, all patients in our department with planned coverage of chronic ulcers or large skin defects in the lower legs, with STSGs from the scalp, were asked to participate and were recruited prospectively.

Detailed patient information, including demographic characteristics and defect-related data (such as the cause of the defect, diameter, percentage of grafting success, and the presence of complications), was systematically collected.

The most pertinent findings are summarized in Table 1. Subsequently, a comprehensive descriptive analysis was performed.

The study received approval and was monitored by the institutional review board of the University Clinic of Navarra.

### 2.2. Study Methodology

Donor areas consisted of sideburns, including its extension to the hairy temporal skin (9) and the hairy occipital skin (6). The donor area was shaved and local anesthesia with 2% mepivacaine was given superficially. For small grafts, we shaved either the left or right parieto-occipital region, and in the case of larger grafts, both regions were utilized. Narrow strips of thin skin (approximately < 0.2 mm thick) were harvested using the disposable, commercially available DermaBlade. The blade was positioned almost parallel to the skin surface and moved gently back and forth while advancing shallowly into the superficial dermis, taking care not to damage the follicular roots.

Areas of approximately 2 mm of healthy hairy skin were intentionally left between each of the donor sites to promote the process of epithelialization. The management of the donor site is a critical consideration, since patients often report more discomfort at this location compared to the recipient site. In our series, we meticulously attended to the donor areas to prevent complications, including bleeding, infection, and the development of noticeable scarring.

After obtaining the skin grafts, we ensured rigorous hemostasis of the scalp through different modalities, such as applying Monsel solution, aluminum chloride 20–35%, ferric subsulfate 20%, or 30% trichloroacetic acid [20]. These substances were placed onto the exposed dermal tissue with a cotton-tipped applicator using a rolling or twisting motion. Superficial electrocoagulation was also performed using a low potency electric scalpel. Finally, a paraffin gauze dressing was placed over the donor site to promote faster healing and epithelization and to relieve pain. We refrained from using antibiotic ointments under the petrolatum dressing as they have not been shown to decrease the risk of infection and may even increase the risk of developing contact dermatitis [18].

The wounds were covered with a dressing for 24 to 48 h or until wound exudate accumulated. Then, the dressing was carefully removed and the lesions were cleaned with a foam wash using movements without friction. Finally, they were properly dried with a sterile gauze or even with a hair dryer and impregnated with povidone–iodine solution twice a day. This procedure was performed daily until complete healing of the donor site, which usually occurred in an average of 12 days. In our experience, wearing the wound uncovered reduces the production of exudate and favors epithelialization. Furthermore, the immobility of the scalp area does not impede mobility and lowers the risk of bleeding, eliminating the use of a dressing.

**Figure 1:**
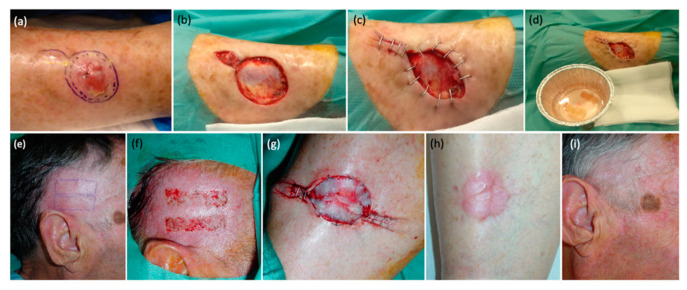
STSG skin grafting procedure using DermaBlade ((**a**–**d**) and (**e**–**i**) correspond to different patients). (**a**) Basal cell carcinoma in the lower leg. (**b**) Final defect after complete tumor excision. (**c**) Final defect size after employing staples to reduce the dimensions of the wound. (**d**) Strips of ultra-thin skin-obtained graft previously submerged in diluted antibiotic (cephalexin and sodium chloride 0.9%) to be placed over the defect. (**e**) Shaved hair from the temporal region as the donor site. (**f**) Scalp donor site (hairy temporal skin) immediately after obtaining ultra-thin strip STSGs with DermaBlade. (**g**) Grafts were placed and sutured in the defect (staples were also used). (**h**) Completely epithelialized wound on a leg one-month post-procedure. (**i**) Completely epithelialized donor site without scarring and with hair regrowth, one-month post-procedure.

Relative to the grafts, the harvested strips were submerged in diluted antibiotic solution (cephalexin and sodium chloride 0.9%) and were carefully placed over the receptor area, covering at least 75% of the defect surface. All grafts were meticulously extended to prevent the formation of wrinkles that could potentially hinder their adhesion. When the defect was due to a surgical excision performed immediately before, the receptor area was already prepared, while in cases of chronic ulcers, a prior careful refreshment of the bed and edges was performed with a scalpel. In some cases, staples or simple sutures were utilized to reduce the size of the defect, to minimize the required number of STSGs, to reverse the edges, and to facilitate the epithelialization process (Figure 1). Sutures were not needed to fix the strips in most cases, as grafts naturally adhered to the recipient area with the assistance of a compressive dressing, obviating the need for suture material. Nevertheless, in specific cases such as long-standing ulcers, elderly patients, and defects situated adjacent to joints, suture material was employed. We used silk in six cases (silk 5/0), which were removed after 5 days. This was done to minimize the risk of graft displacement and enhance their viability. Finally, a compressive wound dressing with petrolatum gauze was applied over the strips, ensuring that it was properly secured to prevent displacement, as commented above. All patients were able to move freely and were discharged on the same day of the surgery. Relative rest was advised for 1 day, and pressure dressings were left in place for 72 h. Then, the dressing was carefully removed. In some cases, it could remain uncovered from this moment on and in some patients, we applied a simple dressing in order to avoid trauma and manipulation for a few more days. Complete healing usually occurred in an average of 24 days.

## 3. Results

A total of 15 patients with a median age of 72 years, including 8 men and 7 women, were prospectively recruited. The series consisted of defects from different etiologies all located in the lower legs: venous ulcers (4), squamous cell carcinomas (6), basal cell carcinomas (2), melanoma in situ (1), nephrocalcinosis (1), and a rheumatic ulcer (1).

The procedures were performed under local anesthesia, in most cases in the same setting as the tumor removal (in the cases of carcinomas and melanoma in situ) and in patients with ulcers, neprhocalcinosis, and rheumatic ulcers, it was specifically scheduled.

The defects varied in size, ranging from 4 cm^2^ to 98 cm^2^ (median: 27 cm^2^). All patients were discharged on the same day as the procedure and none required hospital admission. The entire series of patients were prescribed oral antibiotics (amoxicillin clavulanic) to be taken at home for a whole week and no wound infections were reported. Grafts successfully anchored in 13 out of 15 patients (90%) and no significant postoperative complications were observed. Only one patient with a basal cell carcinoma and another with a rheumatic ulcer experienced partial graft loss, which later healed completely with local treatments without needing a second surgery. These isolated complications were probably related to the formation of a hematoma under the grafts and to involuntary displacement movements that made it difficult for them to take hold. The median time for re-epithelialization was 12 days for the donor site and 24 days for the receptor site. No transfer of hair to the recipient areas or occurrence of alopecic scarring in the donor sites were observed. Finally, after a year of follow-up, there were no instances of ulcer or tumor recurrence.

## 4. Discussion

The practice of skin grafting has a rich history that spans several centuries. One of the earliest recorded instances of skin grafting occurred over 3000 years ago in India, where skin grafts from the gluteal region were used to reconstruct amputated noses as a form of punishment. It was not until the 1800s that skin grafting was widely accepted as a safe and effective treatment for wound management, and shortly thereafter for burn injuries. In the 19th century, pioneers like Reverdin and Thiersch introduced the technique of punch grafting for wound coverage [1,4]. Towards the end of the century, Ollier and Thiersch described the technique of STSGs, and during the same period, Wolfe and Krause introduced methods for full-thickness grafting [1,20]. These historical milestones have laid the foundation for the advancement and refinement of skin grafting techniques that continue to be used in modern medicine. 

Traditionally, two types of grafts are utilized for the treatment of chronic venous ulcers: STSGs, also named Thiersch- or Padget-type grafts, and punch grafts or Reverdin-type grafts [1,5]. Each type has its own advantages and disadvantages, which influence their suitability for the specific areas requiring treatment. Chronic ulcers, particularly those found on the lower limbs, often have a poor blood supply and a fibrous base, making thick split- or full-thickness grafts prone to rejection [1]. Despite offering better protection for the wound, thick grafts are not ideal for these conditions. However, punch grafts, also known as Reverdin grafts, can be suitable for treating chronic leg wounds [21]. These grafts have a higher likelihood of successful engraftment compared to other types and can survive in ulcers with severe infection or limited blood supply. Moreover, by placing punch grafts as close together as possible, the final outcome can be comparable to that of a thick split- or full-thickness graft. Indeed, the punch graft technique offers the advantage of simplicity, requiring minimal surgical instruments and enabling quick outpatient procedures [1]. However, when compared to STSGs, punch grafts have notable disadvantages. Treating extensive lesions with punch grafts can be time-consuming, as it involves cutting and placing numerous small grafts. In contrast, thin STSGs obtained using calibrated knives or dermatomes can cover larger surface areas when cut into long strips [22]. Additionally, the healing process for punch-grafted ulcers tends to be slower compared to split-grafted ulcers. This is because the areas between the small punch grafts fill with scar tissue, necessitating several weeks for complete healing [23]. As an alternative to punch grafts, Vilalta et al. [23] suggested the use of free laminar thin-band grafts obtained with a 23 blade scalpel, further emphasizing the benefits of thin STSGs in certain cases. We have observed certain limitations in using scalpel blades to obtain STSGs. This method lacks reproducibility and makes it challenging to predict the thickness of grafts accurately. Additionally, obtaining thin and long grafts can be difficult, and there is a risk of inadvertently cutting or damaging the reticular dermis or the adipose tissue, which may lead to scarring [24,25].

Another significant contribution to the field of reconstructive surgery was the development of the dermatome, a surgical instrument widely employed for skin grafting. In fact, its primary purpose is to create thin, uniform skin grafts from a donor site, which can subsequently be transplanted to treat various cutaneous defects, particularly those of a significant size. One of the key advantages of the dermatome is its ability to procure large, uniform grafts efficiently, offering the advantage of precision and allowing for calibration to achieve the desired graft thickness. Remarkably, a single pass of the dermatome is often sufficient to harvest an appropriate graft size. These features make it an indispensable tool in the armamentarium of reconstructive surgeons. Nonetheless, it is imperative to acknowledge that mastering the dermatome requires a substantial learning curve, and consistent and high-quality results hinge on the experience and skill of the surgeon. Inexperienced operators may encounter challenges such as uneven graft thicknesses or fragmentation, potentially compromising graft success. In addition, a recognized complication associated with dermatome use is dermatome-induced laceration. Several factors contribute to this complication, including patient-specific variables such as skin laxity and atrophy or surgeon factors such as errors in blade calibration, where the dermatome is not properly set to the desired thickness. Furthermore, both the speed and the pressure applied during the process of graft extraction play a pivotal role in determining the risk of laceration [25].

**Figure 2:**
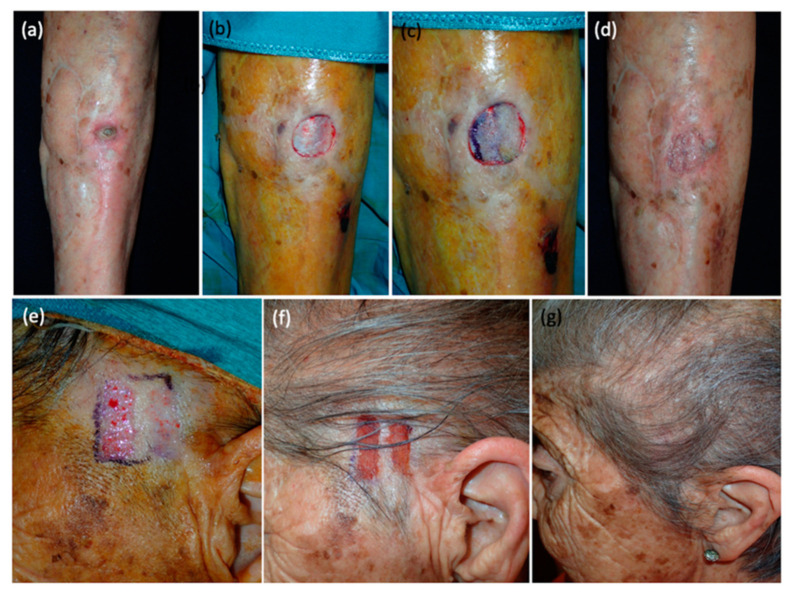
Completely epithelialized squamous cell carcinoma after grafting STSG with DermaBlade. (**a**) Squamous cell carcinoma in the lower leg. (**b**) Immediate defect appearance after complete tumor excision. (**c**) Grafts are placed in the defect with no sutures. (**d**) Completely epithelialized wound on the leg, one-month post-procedure. (**e**) Scalp donor site (hairy temporal skin) immediately after obtaining ultra-thin strips with DermaBlade. (**f**) Scalp donor site (hairy temporal skin) days after obtaining the strips. (**g**) Completely epithelialized donor site without scarring and with hair regrowth, one-month post-procedure.

**Figure 3:**
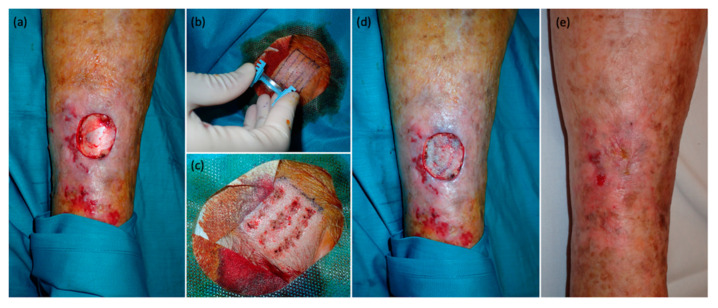
Completely epithelialized squamous cell carcinoma defect after using STSG taking from the scalp with DermaBlade. (**a**) Immediate defect appearance after complete excision of squamous cell carcinoma. (**b**) Use of DermaBlade to harvest STSG from the hairy sideburn area. (**c**) Scalp donor site (hairy temporal skin) immediately after obtaining ultra-thin strip STSGs. (**d**) Grafts are placed in the defect with no sutures. (**e**) Completely epithelialized wound on the leg, one-month post-procedure.

**Figure 4:**
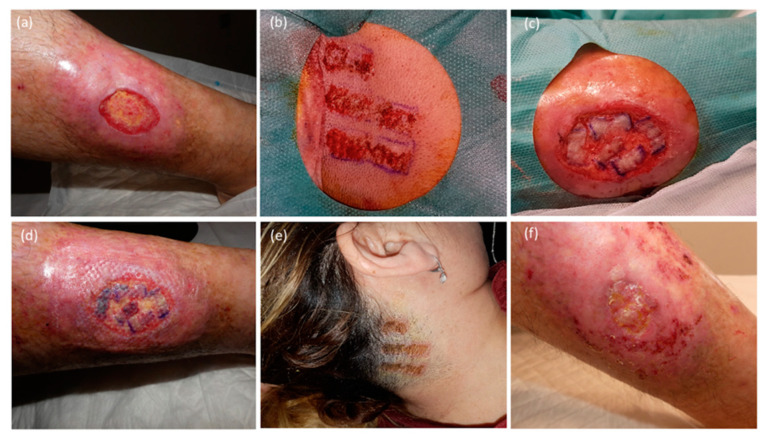
Completely epithelialized venous ulcer after using STSG removal from the scalp with DermaBlade. (**a**) Recalcitrant venous ulcer defect. (**b**) Scalp donor site (hairy occipital skin). (**c**) Grafts are placed in the defect (consequently, a compressive wound dressing was applied without suturing). (**d**) Receptor area after 3 days. (**e**) Donor occipital area after 10 days. (**f**) Ulcer aspect after 28 days.

In our experience, the use of the DermaBlade cutting tray has provided a solution to these challenges, particularly relevant regarding narrow and thin strip grafts [26,27,28]. This tool is easy to use, enhances reproducibility, allows for better predictability of graft thickness, and facilitates the extraction of thin, uniform, and different length grafts [29]. By mitigating the risks, the DermaBlade cutting tray improves outcomes and preserves the advantages of using STSGs. To be precise, the DermaBlade enables the harvesting of STSGs, allowing for thin grafts (<0.2 mm) composed of just the epidermis and the superficial papillary dermis. As previously reported, it is crucial to obtain grafts with a thickness of less than 0.2 mm, as there is evidence to suggest that a thickness exceeding this measurement can lead to visible scarring and alopecia in the case of scalps [17]. Moreover, these thin grafts adhere to the wound bed within 2–3 days, often without the need for stitches, as demonstrated in our series (Figure 2, Figure 3 and Figure 4).

Regarding the choice of the donor site, the use of skin grafts harvested from the scalp was first introduced by Crawford and has been reported by numerous authors since then [8,9,10,17,30,31]. The selection of hairy skin as a donor site offers several advantages, including rapid wound healing, a low risk of complications, and excellent cosmetic results without visible scarring or alopecia, making it a viable choice as a donor site. The scalp possesses notable epidermal repair capabilities, remains relatively immobile, and offers the advantage of scar concealment through hair growth (camouflage effect) [32].

In addition, the enhanced healing process is attributed both to the abundant presence of follicular epithelial stem cells and the rich vascularity of the scalp, which endows it with the capacity for rapid re-epithelialization in superficial layers and the synthesis of collagen and elastin in deeper layers [12,33,34]. Hair-follicle-derived cells play the role of a wound-healing promoter in epidermal wound closure. After an injury, the regeneration of the epidermis involves several processes, including the activation, migration, and proliferation of keratinocytes from the surrounding epidermis and adnexal structures such as hair follicles and sweat glands. Notably, the follicular epithelium plays a significant role in the initial resurfacing of the wound. Moreover, cells derived from the follicular epithelium remain within the basal layer of the epidermis and serve as long-term repopulating cells responsible for maintaining the regenerated epidermis in the wound area. Through effective lineage conversion, follicular stem cells can assume the function of resident epidermal stem cells, and those located in the bulge region of hair follicles are considered the ultimate source for generating all cutaneous epithelial lineages [9]. Furthermore, there are articles that contain comparisons between the scalp and the thigh as donor sites for STSGs, confirming this theory and remarking the superiority of the scalp. These studies argue that the scalp offers advantages that result in fewer side effects, while providing a comparable graft quality to those grafts harvested from the thigh [15].

Complications derived from having the scalp as a donor site are unusual but exist, and include mainly scab formation, erythema, and wound infections. Scab formation typically resolves within a few weeks after surgery, while erythema may gradually improve over a period of even a year, depending on the depth of the wound. On the other hand, wound infections and folliculitis, if present, need to be properly managed and necessitate aggressive treatment with sensitive antibiotics and topical wound care. Uncontrolled complications in this category can lead to alopecia or if severe, to tufted scar deformities [17]. In our series, no instances of wound infection or folliculitis occurred. This favorable outcome may be due, in part, to the systematic administration of oral antibiotics. Additionally, the scabs and erythema presented within the donor area gradually dissipated in the weeks following the intervention thanks to the meticulous management and wash detailed in the Material and Methods section.

Herein, we included a series of patients with diverse lower extremity defects, including challenging and slow-healing conditions such as venous ulcers, rheumatic ulcers, and nephrocalcinosis with extensive areas requiring coverage of up to 98 cm^2^ (Figure 4). Given the complexities associated with these cases, the use of STSGs was deemed more appropriate than punch grafts, based on the reasons previously discussed. Moreover, as mentioned above, we attribute the positive results observed in our study to three key innovations.

Firstly, the ultra-thin nature of the STSGs facilitated a better adherence of the strips, enhancing their viability and enabling wound epithelialization, even in elderly patients with chronic ulcers and multiple comorbidities. Furthermore, the thickness of the grafts less than 0.2 mm ensured the absence of scarring and alopecia in the donor areas. Secondly, the adoption of the DermaBlade cutting tray for graft extraction instead of a conventional scalpel promoted the rapid and effortless procurement of superficial, ultrafine, consistent, and highly viable grafts that adhered to the wound bed in 2 or 3 days [8,9,12,13,14,15,16,17,35,36]. In our experience, compared to traditional STSGs, using STSGs obtained with DermaBlade led to significant advantages. Notably, they often do not necessitate a tie-over dressing, suturing was not required at the edges of the wound in most cases, and the initial dressing can be removed after 72 h instead of the standard 5 days. Moreover, in our series, these grafts exhibited a high efficacy and viability, with successful engraftment in the recipient areas in all cases. Third and finally, we consider that the selection of the hairy scalp skin as the donor site played a crucial role. As previously discussed, we believe that the abundant vascularity in this region, along with the presence of hair follicles and follicular stem cells between the graft extraction sites, significantly contributed to the swift epithelialization of the donor area and the absence of complications, enhancing the satisfaction of patients and delivering optimal healthcare conditions [9,37].

### Limitations

Despite the effort of the authors to mitigate them, this study has some limitations. First, the small sample size could limit the generalizability of the findings. In addition, although the technique demonstrated a high efficacy rate of 90% for graft success with relatively short healing times for both the donor and recipient areas, these results were not compared to an alternative treatment or control group. While other studies have previously conducted comparisons between patients utilizing scalp skin and thigh skin as donor areas, our series exclusively employed the scalp due to its well-established advantages. Finally, all the strips were submerged in a cephalexin solution and after the procedure, oral antibiotic prophylaxis was used in all patients to minimize the risk of infection. Although we obtained satisfactory results, we cannot probe the real role or need for antibiotics as we did not compare results with a control group without them.

## 5. Conclusions

Herein, we present a series of patients with lower limb wounds from different etiologies, including surgical defects and ulcers that were reconstructed with ultra-thin STSGs obtained from the scalp with DermaBlade. As has been demonstrated, it is a simple, fast, and easily reproducible technique with no mobility limitations that requires just a few pieces of surgical equipment and allows rapid donor site and defect healing, with great viability, low risk of complications, and excellent cosmetic results. We consider these two innovations, namely the utilization of the DermaBlade for graft extraction and the choice of the scalp as the donor site, to be pivotal in the successful outcomes and positive results observed in our study. Further additional prospective and comparative studies, including more extensive series, would be valuable to validate our results and to explore innovative grafting techniques that can enhance the reconstructive possibilities for lower extremity defects.

## Figures and Tables

**Table 1:** Information on the series, including demographic characteristics of patients and defect-related data.

Patient	Gender	Age	Cause	Location	Size (cm^2^)	Donor Site	Suture	Complications	Recurrence *
1	F	65	VU	RL	22	TS	NO	NO	NO
2	F	72	SCC	RL	4	TS	NO	NO	NO
3	M	58	MS	LL	22	OS	NO	NO	NO
4	F	76	SCC	LL	46	TS	YES	NO	NO
5	F	83	SCC	LL	12	OS	YES	NO	NO
6	M	64	RU	RF	52	OS	NO	YES	NO
7	M	69	BCC	RL	98	TS	NO	YES	NO
8	F	77	SCC	LF	8	TS	YES	NO	NO
9	M	53	NFC	RL	12	OS	NO	NO	NO
10	M	88	VU	RL	8	OS	NO	NO	NO
11	F	75	SCC	LL	32	TS	NO	NO	NO
12	M	70	BCC	LL	40	TS	YES	NO	NO
13	M	86	VU	LL	16	OS	NO	NO	NO
14	F	87	SCC	RL	18	TS	YES	NO	NO
15	M	59	VU	LL	20	TS	NO	NO	NO

Abbreviations: F = female; M = male; VU = venous ulcer; SCC = squamous cell carcinoma; RU = rheumatic ulcer; BCC = basal cell carcinoma; MS = melanoma in situ; NFC = nephrocalcinosis; RL = right leg; LL = left leg; RF = right foot; LF = left foot; TS: temporal skin; OS = occipital skin. * Recurrence was evaluated after a year of follow-up.

## Data Availability

Data supporting reported results can be found by contacting to the corresponding author.
